# Supplementary material for: Are carbon emissions trading and green financial instruments synergistic? -Comprehensive quantitative research based on content analysis
Source: PLoS One. 2024 Mar 7;19(3):e0298601. doi: 10.1371/journal.pone.0298601 (PMC10919601; doi:10.1371/journal.pone.0298601)
Supplement: S1 Data — (ZIP) [file pone.0298601.s001.zip › Data/地级市Z_F工作报告.zip!/║■▒▒/╧╠─■╩╨/└┤╫╘╣1⁄2╓┌║┼í╛┬φ┐╦╩2╛▌═°í┐_2013.docx]

各位代表：

现在，我代表市人民政府，向大会报告政府工作，请各位代表审议，并请市政协委员和其他列席人员提出意见。

一、2012年工作回顾

刚刚过去的一年，在省委、省政府和市委的坚强领导下，在市人大、市政协和社会各界的监督支持下，市政府紧紧依靠全市人民，深入贯彻落实科学发展观，着力扩内需、调结构、稳增长、惠民生，市四届人大一次会议确定的当年目标任务圆满完成。初步预计，全市生产总值753亿元，增长12%，增加额连续三年超过100亿元；地方公共财政预算收入45.8亿元，增长28.9%。

一年来，我们主要做了以下工作：

（一）有效应对复杂形势，经济平稳较快增长。切实抓好各项稳增长政策的落实，加强经济运行分析和调度，加大解决突出问题工作力度，在全市上下营造抢机遇、争项目、促内需的浓厚氛围。全社会固定资产投资730亿元，增长30%。武深高速咸宁段、武汉城市圈环线高速公路咸宁西段开工建设，红牛饮料三期、黄鹤楼酒业、中美厚福医疗器械建成投产，华润（赤壁）电厂二期实现并网发电，武咸城际铁路、咸通高速、通界高速和咸宁南玻玻璃产业园、咸安生态环保产业园、嘉鱼洁丽雅产业基地、赤壁光谷产业园、通城宝塔研磨、崇阳兴民钢圈、通山科奥医疗器械工业园等项目加快推进。社会消费品零售总额280亿元，增长16%。居民消费价格上涨2.8%，物价上涨势头得到有效遏制。

（二）加快推进产业调整，转型升级步伐加快。全市三次产业比重调整为18.1：47.8：34.1。规模以上工业企业674家、净增54家，其中年产值过亿元以上企业310家、增加51家；规模以上工业增加值340亿元，增长17%。各类开发区和工业园区快速发展，嘉鱼管材纳入全省重点成长型产业集群，“麻塘”、“白练及图”获评中国驰名商标。粮食生产实现“九连增”，绿色农产品基地面积355万亩，建成畜禽养殖小区210个，年产值5000万元以上农业产业化龙头企业54家。咸安区获评全国农田水利基本建设先进单位。全市旅游接待人数2100万人次，旅游总收入107亿元，分别增长39.8%、38.4%。成功举办第四届国际温泉文化旅游节，与青岛、苏州、丽江一道跻身首批全国旅游标准化示范市。实施重点节能减排工程，省定节能减排任务圆满完成。

（三）不断加大统筹力度，城乡区域协调发展。大力推进“六城三区”建设，市区一体化进程加快。十六潭公园、青龙山公园一期、嫦娥公园二期和市竹博览馆建成开放，市规划展览馆被命名为“全国科普教育基地”，“多城同创”取得重要进展。加强县市城区建设，嘉鱼环湖公园全面建成，赤壁生态新城、通城樊牌新城建设进展顺利，崇阳“一河两岸”城市景观工程开工建设，通山高标准完成次中心城市总体规划和专项规划。统筹推进城乡一体化，涌现出一批村企合作共建典型。“北三县”沿江产业带和“南三县”生态特色产业带竞相发展，赤壁县域经济综合排名名列全省第19位，重返第一方阵。

（四）着力深化改革开放，创新活力显著增强。“两型”社会建设综改试验取得新进展，“厅市合作”、“校市合作”不断深化。52个市直单位完成企业脱钩改制。新登记各类市场主体29727户，增长33.95%。财税库银横向联网、营业税改征增值税试点工作稳步推进。农村义务教育化债工作全部结束。争取国土整治项目42.3万亩，净增耕地5.2万亩。城投、交投、农投、职教投等融资平台创新发展，示阳农牧股权首开我市企业在天交所挂牌交易先河。新引进武汉农村商业银行、吉林九台农村商业银行，金融机构新增贷款84.4亿元，同比多增35.4亿元。医药卫生体制改革不断深化。文化体制改革受到全国表彰。大力发展开放型经济，引进新开工工业项目509个，招商引资到位资金310亿元，增长56.2%；实际利用外资2.12亿美元，增长20%；完成外贸出口2.33亿美元，增长0.4%。岳九咸“小三角”率先启动。全省首个科技成果转化示范基地落户咸宁。

（五）重视发展社会事业，公共服务继续加强。推进各类教育协调发展，城乡义务教育一体化试点工作在赤壁启动，湖北科技学院更名揭牌，“九年一贯制”浮山中小学建成开学。增强城市文化软实力，市博物馆建成开放，全市“三馆一站”全部实现免费开放。群众性精神文明创建活动不断深入，全民健身活动蓬勃开展。社会科学、文学艺术、新闻出版、广播影视事业加快发展。基层医疗卫生服务体系进一步健全，基本公共卫生服务均等化进程加快，重大传染病预防控制工作成效显著。低生育水平保持稳定，出生人口性别比稳步下降。国土资源保障发展的能力增强。加强国防动员和后备力量建设，双拥共建活动取得新成效。社区信息化建设步伐加快。支援新疆、建始县工作在创新中扎实推进。民族、宗教、对台、外事、侨务工作取得新成绩，老龄、青年、妇女、儿童、科普、慈善、助残事业取得新进步，人事、编制、审计、统计、档案、方志、保密、气象、地震、水文、人防等工作进一步加强，政策咨询、社会团体、驻咸机构在推动经济社会发展中发挥了重要作用。

（六）更加注重以人为本，民生质量持续改善。城镇居民人均可支配收入16800元，增长13%；农民人均纯收入7500元，增长13.8%。新增城镇就业4万人，城镇登记失业率3.83%。全市五项基本社会保险参保157.85万人，城乡居民养老保险实现全覆盖。城乡低保动态管理新增9766户、15051人。新开工建设保障性住房15109套，竣工9100套，发放廉租住房租赁补贴3150户。完成60个贫困村扶贫整村推进年度任务，实现减贫目标4.3万人。幕阜山连片特困地区被列入全省新十年扶贫攻坚主战场。深入推进“平安咸宁”建设，社区网格化服务管理运转良好，咸宁荣获“中国城市管理进步奖”。市政府年初承诺的“十件实事”基本完成。

（七）加强政府自身建设，发展环境得到优化。自觉在市委的领导下履行法定职责。自觉接受人大法律监督、工作监督和政协民主监督，共办理人大议案建议88件、政协提案148件。科学民主依法决策机制不断完善。依法治市和法治咸宁建设有力推进。深化行政审批制度改革，市级行政许可项目减少到93项；市行政服务中心获ISO9000质量管理体系认证。厉行“三短一简”，“治庸问责”力度加大。政府系统党风廉政建设责任制得到有效落实。

过去一年的成绩来之不易，这是中央和省委、省政府正确领导的结果，是市委审时度势、科学决策的结果，是全市人民齐心协力、顽强拼搏的结果。在此，我代表市政府向全市广大干部群众和各位人大代表、政协委员、各民主党派、工商联、无党派人士和离退休老同志，表示诚挚的感谢！向驻咸解放军、武警和消防官兵、政法干警，表示诚挚的感谢！向驻咸单位和在咸投资创业的企业家，以及所有关心支持咸宁发展的各界人士，表示诚挚的感谢！

回顾一年的工作，我们清醒地认识到，我市经济社会发展中还存在不少困难和问题。主要是：经济总量不大、结构不优、质量不高，转方式、调结构任重道远；工业实力偏弱，自主创新能力不足，中小企业融资困难；中心城区辐射带动力不强，县域经济发展不平衡的问题依然存在；城乡居民收入不高，社会管理和公共服务体系还不够完善；少数部门效能不高、执行不力、作风不实的问题仍较突出，政务环境仍需优化，等等。我们一定以对党和人民高度负责的精神，采取更加有力的措施，切实解决存在的问题，努力把咸宁的事情办得更好，决不辜负全市人民的重托与期待。

二、2013年主要任务

2013年是全面贯彻落实党的十八大精神的开局之年，是实施“十二五”规划承前启后的关键之年，是为全面建成小康社会奠定坚实基础的重要一年，做好今年的工作意义重大。今年政府工作的总体要求是：高举中国特色社会主义伟大旗帜，以邓小平理论、“三个代表”重要思想、科学发展观为指导，全面贯彻落实党的十八大、中央和全省经济工作会议、市委四届二次全会精神，紧紧抓住“黄金十年”重要战略机遇，牢牢把握“绿色GDP”和“民生GDP”两个要点，按照稳中竞进、进中求好的总基调，坚持主题主线，深化改革开放，突出创新驱动，促进社会和谐，加快推进省级战略咸宁实施，千方百计做大经济总量，奋力推进科学发展、跨越式发展，为全省“竞进提质”和全国“稳中求进”大局作出新的贡献。

全市经济社会发展的主要预期目标是：地区生产总值870亿元；规模以上工业增加值410亿元；全社会固定资产投资910亿元；居民消费价格涨幅控制在4 %以内；社会消费品零售总额325亿元；地方公共财政预算收入55亿元；外贸出口总额2.64亿美元；实际利用外资2.24亿美元；城镇居民人均可支配收入18980元，农民人均纯收入8480元；城镇登记失业率控制在4.6%以内；人口自然增长率控制在6.5‰以内；全面完成省定节能减排目标任务。

上述预期目标是基本要求，必须确保完成。在实际工作中，要以“跳起来摘桃子”的精神，确定更加积极的工作目标，力争更好的结果。为此，重点做好以下五个方面的工作。

（一）坚定不移地推进工业崛起。工业是财富之源、发展之基，是咸宁能否打翻身仗的关键所在。我们将增强忧患意识、危机意识，强化工业主体地位不动摇，排除一切干扰，凝聚一切力量，克服一切困难，全力推进工业经济超常规、跨越式发展。

做大做强做优产业。大力实施“三个百亿”产业工程，扎实推进咸宁南玻玻璃产业园、湖北（咸宁）广东工业园、咸宁生物医药产业园、华帝燃具、华科创新基地和咸安模具产业园、嘉鱼金盛兰特种钢和扬子江汽车、赤壁宇顺电子和德力西林浆纸一体化、通城亚细亚陶瓷、崇阳兴民钢圈、通山武钢新辅料基地等一批重点项目，加快培育一批百亿元大产业、百亿元大企业和百亿元大园区。继续推进电力能源、冶金建材、森工造纸、纺织服装、机电制造、食品饮料等六大支柱产业向产业链两端延伸，积极培育新能源、新材料、高端装备制造、新一代信息技术、节能环保、生物医药等战略性新兴产业。加快推进汽车零部件产业发展，建设咸宁汽车零部件产业园。进一步做好咸宁核电、大湖山风力发电等项目前期工作。

做大做强做优园区。全力推进咸宁经济开发区加快发展，围绕打造100平方公里武咸工业走廊，调整建设一期、全面建设二期、启动建设三期，力争实现每月开工项目4个、投产项目4个、签约项目4个的“三个四”目标，继续做好创建国家级开发区工作。支持县（市、区）抓好新型工业化示范园区建设，形成各具特色的支柱产业和特色产业集群。坚持“一张蓝图画到底”，优化园区功能分区，完善园区基础配套，加强园区闲置用地清理、建设用地控制，构建“产城融合、园城一体”的发展格局。

做大做强做优企业。支持骨干企业通过技术改造、兼并重组、合资合作等形式做大做强，培育形成一批行业领军企业。扎实推进“市场主体增量行动”，确保新增规模以上工业企业100家。优先扶持厚福医疗、能一郎电池、瀛通电子等具备条件的企业做好上市工作。加大名牌产品创建力度，新增中国驰名商标2个以上。落实结构性减税、信贷倾斜等优惠政策，促进小微企业健康发展。加强煤电油气运等要素供给，保证企业正常生产经营。

做大做强做优投资。加强重大项目的谋划、储备、推进、申报和衔接，实行“一月一检查、一季一拉练、一年一总结”，保持投资较快增长。加快推进以武深高速咸宁段、武汉城市圈环线高速咸宁东段和西段、咸通高速、通界高速、嘉鱼长江大桥、赤壁长江大桥、咸潘一级公路、潘家湾码头、咸宁南外环高速等项目为重点的基础设施建设工程，确保武咸城际铁路年内全面开通。进一步抓好省委、省政府承诺的4大类30个重大项目的跟踪衔接，确保早见成效。落实鼓励和引导民间投资的政策措施，进一步扩大民间投资。

促进二、三产业协调发展。着力打造金融保险、商贸物流、网络信息、服务外包等现代服务业集聚区。支持发展新合作鄂南物流园、咸安商贸物流园、嘉鱼临江物流园、赤壁沿国道物流集聚区、通城边贸物流中心、崇阳天城物流园、通山石材物流中心，积极引进国际国内大型商贸企业落户咸宁。推进百盟欢乐大世界、温泉谷二期、太乙地质公园、桂花源、桂香源、咸宁自驾游基地、浪口温泉二期、通城生态旅游、幕阜山旅游公路等项目加快建设，力争接待游客突破2800万人次。继续办好国际温泉文化旅游节。大力发展移动互联网、云计算、物联网等新兴产业，推进“数字（智慧）咸宁”建设。做好全国家政服务试点城市工作，争取列入国家再生资源试点城市。

（二）坚定不移地推进改革开放。改革开放是发展的最大“红利”，是建设幸福美好新咸宁的必由之路。我们将以更大的勇气和智慧，坚决破除一切妨碍科学发展的体制机制弊端，加快形成敢开放、真开放、先开放、全开放的生动局面。

进一步深化重点领域改革。深入实施武汉城市圈“两型”社会建设综合配套改革。健全国有资产监管体制。加强财政科学化、精细化管理，完善公共财政体系。深化收入分配制度改革，推进企业和机关事业单位工资制度改革。加强政府债务管理和化解，规范投融资平台建设。理顺住房公积金管理体制。完善金融监管，推进金融创新。加快推进要素配置市场化改革，促进各类要素汇聚咸宁。深化农村综合配套改革，推进扩权强镇改革试点。稳妥推进户籍制度改革。完善公务员选用和管理机制。加快推进事业单位分类改革。

进一步扩大开放合作。坚持招商与“选商”相结合，注重招大项目、招好项目，加强与央企的对接合作，争取战略投资机构落户咸宁。落实稳定出口政策，加强出口基地建设，培育壮大一批出口龙头企业。继续做好海关、商检咸宁分支机构设立工作。加强与珠三角、长三角、环渤海、武汉城市圈、长株潭城市群、环鄱阳湖生态经济区的经济联系，深化岳九咸“小三角”区域合作。加强与海外友好城市的往来，积极开展人力资源领域的国际交流与合作，提升咸宁国际化水平。

进一步推进创业创新。牢固树立“产业第一、企业家老大”的理念，培育重商爱企的发展文化。毫不动摇地鼓励、支持、引导非公有制经济发展，扶持壮大民营企业。实施“香城创业计划”，全面推进国家级创业城市创建工作。完善创业扶持政策，健全创业服务体系，推进全民创业，实现新增个体工商户1万家以上。鼓励和引导企业加大技改和研发力度，提升企业核心竞争力。加强省科技成果转化基地建设，深化与武汉大学、华中科技大学等高校和科研院所的合作。完善人才引进、培养、激励机制，着力引进科技领军人才，培养一线创新人才。

（三）坚定不移地推进城乡统筹。统筹城乡发展，是科学发展观的重要内容，也是全面建设小康社会的重大任务。我们将加大统筹城乡发展力度，把“四化同步”作为解决“三农”问题的根本途径，整体推进城乡一体化发展，促进城乡共同繁荣。

大力发展现代农业。认真落实各项强农富农惠农政策，加大高产农田、板块基地建设和农业综合开发力度，加快推进以农田水利为重点的农村基础设施建设，改善农业生产条件，确保全年粮食生产稳定发展。加大农业产业化龙头企业、农业专业合作社、农业专业大户等农业主体培育力度，鼓励创新农村土地经营机制，提升农业产业化经营水平。围绕打造百亿元竹产业、百亿元茶产业，全面落实竹产业发展“十三条”，打造楠竹产业基地；支持赤壁发展砖茶产业园，支持通山发展瑶山红茶，重振咸宁茶叶雄风。大力发展标准化生产，新增“三品”认证30个、国家地理标志认证1个。加快构建农村社会化服务体系，大力推进新型庄稼医院建设，重点解决农技推广“最后一公里”的问题。

大力推进新型城镇化。规划实施“香城泉都•浪漫咸宁”主题活动，推进“全域香城”建设，促进“泉都”提档升级。加快中心城区建设，完善城市供水、排水、污水处理、垃圾处理、供气、供电、公交、通讯、道路、水系整治、园林绿化等公共基础设施建设，持续推进创建国家文明城市、国家卫生城市、国家环境保护模范城市等“多城同创”工作。全面加强城镇规划建设和管理，做到精心规划、精致建设、精细管理。做好城市规划区个人住宅规划建设和农村宅基地管理工作。统筹推进城乡一体化试点，加快中心镇、中心村、社区建设。以新一轮“三万”活动为抓手，大力开展“十星级文明户”创建工作，扎实推进新农村建设。

大力推进区域协调发展。以咸宁经济开发区为重点，高昂市区发展龙头，推动梓山湖生态新城、职教新城超常规发展。以咸嘉新城为重点，加快长江经济带开放开发，大力推进“一城两园六港”建设。以“一主三化”为重点，支持咸安打造“香城泉都”中心城、构建低碳经济示范区、争当鄂南强市排头兵，支持嘉鱼建设湖北经济强县、打造长江临港新城，支持赤壁建设强而优中等城市，支持通城建设幕阜山区综合开发示范县、打造“中三角”节点城市先行区，支持崇阳建设幕阜山区经济强县、打造美丽富饶新天城，支持通山建设绿色崛起先行区、打造咸宁次中心城市。围绕县域经济主要指标“四年翻番”的目标，支持赤壁冲刺全省县域经济10强，支持咸安、嘉鱼冲刺全省县域经济第一方阵，支持“南三县”争先进位。

大力推进幕阜山片区扶贫开发。认真贯彻落实省委、省政府《关于加快推进幕阜山连片特困地区区域发展和扶贫攻坚工作的意见》，统筹推进政府扶贫、产业扶贫、项目扶贫和社会扶贫。加大政策、资金和项目的支持力度，重点抓好交通、农田水利、能源等基础设施建设，大力发展高效农业、现代工业、生态文化旅游业等产业。建立市级领导联县包村扶贫、“北三县”与“南三县”结对共谋发展、部门与乡镇对口帮扶等机制，把幕阜山片区打造成为脱贫致富的先行区、生态保护与开发的示范区、绿色发展的试验区。

（四）坚定不移地推进绿色发展。咸宁生态环境优良，这是大自然的恩赐，也是前人留下的财富。我们将把生态建设和环境保护摆在更加突出的位置，在实现绿色发展、可持续发展中彰显咸宁的山水优势，让青山常在、蓝天常驻、碧水长流。

加大环境保护力度。扎实推进“生态持续”战略，建设美丽咸宁。扎实抓好退耕还林、天然林保护、长江防护林、生态公益林、低产林改造等林业重点生态工程，确保完成30万亩造林任务。继续抓好“两湖”、“三河”、“十库”和簰洲湾民堤等重点水域流域生态保护与综合治理。加强湖泊水库保护，支持陆水湖纳入全国优良湖泊保护规划纲要。深入推进农村环境连片整治示范工程，重点解决饮用水不安全、空气污染、土壤污染等突出环境问题。积极开展生态镇（村）创建工作。

加大节能减排力度。严格落实节能减排目标责任制，继续实施重点节能减排工程，确保完成今年节能减排任务。大力发展低碳经济、循环经济，加快金桂湖低碳经济示范区、赤壁和咸安等地循环经济产业园建设。深入开展工业园区污染集中治理，加快乡镇污水处理厂建设。做好主要污染物排污权交易试点工作。

加强资源节约集约利用。实行最严格的耕地保护和水资源管理制度。加快推进1.45万亩城乡“增减挂钩”项目建设和2.7万亩低丘缓坡土地综合开发利用省级试点。强化对开发强度、建筑密度、容积率、绿地率等指标控制，保障重点建设项目和民生工程用地需求。加强矿产资源勘查、保护、合理开发。

（五）坚定不移地推进以改善民生为重点的社会建设。加快发展的根本目的就是为了惠及百姓、改善民生。我们将更加重视以改善民生为重点的社会建设，更加关注全市人民的生活质量、发展潜能、幸福指数，努力让全市人民过上更好生活。

努力提高居民收入。按照国家2020年城乡居民人均收入比2010年翻一番的目标，制定居民收入倍增计划，力争今年城镇居民人均可支配收入、农村居民人均纯收入分别增加1850元、830元。加强“米袋子”、“菜篮子”工程建设，保持物价总水平基本稳定。

突出改善民生质量。把扩大就业放在优先位置，统筹做好高校毕业生、农村转移劳动力、就业困难人员和退役军人四类重点群体就业工作。建立完善失业预警和调控机制。以提高统筹层次和待遇水平为重点，健全多层次的社保体系，不断扩大覆盖范围，稳步提高保障水平。加强保障性安居工程建设，改造农村特困家庭危房，改善城乡居民的居住条件。加快建设王英水库引水工程。建立健全城乡一体化社会救助体系和救灾应急体系，统筹做好社会保障、社会救助、社会福利、社会慈善等工作。坚持以公平优质为重点，办好更加规范的学前教育、更加均衡的义务教育、更具特色的职业教育、更高水平的高中教育和高等教育。深化医药卫生体制改革，扩大公立医院综合改革。全面实施城镇居民医保门诊统筹，探索建立职工医疗保险门诊统筹。开展城镇居民大病医疗保险，继续推进医疗保险城乡统筹试点工作，改革医疗保险付费方式。综合治理出生人口性别比偏高问题，稳定低生育水平。发展妇女儿童事业，重视老龄和残疾人工作。加强未成年人保护，关心关爱留守儿童的生活和成长。进一步做好法律服务和法律援助工作。

大力繁荣文化事业。加强社会主义核心价值体系建设，全面提高公民道德素质。大力发展公益性文化事业，扩大文化惠民覆盖面，促进公共文化服务方式的多样化、社会化。积极发展文化产业，促进现代传媒、新闻出版、文化娱乐、体育健身业加快发展。探索文化市场管理的长效机制。加快推进市妇儿活动中心和科技馆、市图书馆和档案馆、向阳湖文化名人展示馆等项目建设。

加强和创新社会管理。加强基层社会管理和服务体系建设，健全社区网格化服务管理。加强和改进新形势下的信访工作，积极排查化解矛盾纠纷，切实维护人民群众合法权益。夯实安全生产基础，落实安全生产责任制。强化食品药品监管。深化“平安咸宁”建设，健全完善社会治安防控体系，高度重视禁毒工作，严厉打击各类犯罪。加强国防动员、国家安全、民兵预备役、人防工作，支持驻咸部队建设。积极创建全国双拥模范城，巩固发展军政军民团结。继续做好支援新疆、建始县工作。进一步加强民族、宗教、外事、侨务、对台等工作。统筹推进消防、气象、水文、地震、档案、方志、保密等工作。

本着普惠、积极、当年的原则，今年继续为城乡居民办好“十件实事”：（1）筹措资金2亿元，在全市909个村开展以“两清”、“两建”、“两化”、“两创”为重点的村庄环境整治活动。（2）新增城镇就业3.5万人，农村劳动力转移就业3万人，城镇失业人员再就业0.6万人，组织城乡劳动者就业、创业培训1.6万人。（3）建成各类保障性住房7360套，其中新建廉租住房390套，建设公共租赁住房4000套，实施各类棚户区改造1670套，新增发放廉租住房租赁补贴1300户；实施7000户农村危房改造工程。（4）解决6万贫困人口脱贫问题。（5）全面推进淦河下游二期综合治理；新解决7万农村人口饮水安全问题。（6）继续推进背街小巷综合治理，基本实现市区路灯全覆盖；新建一批公厕，改造一批旱厕、危旧公厕；增加城区停车泊位。（7）推进义务教育均衡发展，让城乡孩子共享优质教育；改善市城区学校办学条件和道路交通安全；在咸宁经济开发区新建1所“九年一贯制”中小学。（8）筹措资金4000万元，帮扶10000户计生家庭脱贫致富。（9）规划建设市老年人活动中心；为全市80岁以上老年人发放高龄补贴。（10）为7000名符合条件的农村贫困残疾人发放生活补贴，开展0—6岁残疾儿童抢救性康复，为3000名贫困城乡残疾人免费配发辅助器具。

三、努力建设人民满意政府

政府工作的根本宗旨是为人民服务，最高目标是让人民满意。我们将把建设人民满意政府作为始终不渝的理念，贯穿到政府工作的各个环节，真正让政府提升效能、群众得到实惠，形成推动经济社会发展的“正能量”。

坚持对人民负责。严格按照法定权限和程序行使权力、履行职责，切实规范行政执法行为。健全行政决策机制和程序，把公众参与、专家论证、风险评估、合法性审查和集体讨论决定作为重大决策的必经程序，推进决策科学化、民主化、法治化。加快法治政府建设，全面开展依法行政示范单位创建活动。健全执行机制，完善责任体系，构建科学合理的落实链条，推进政府绩效管理。所有政府部门和工作人员都要强化大局意识、责任意识、执行意识，敢于担当、敢于负责、敢抓敢管，确保政令畅通。

坚持为人民办事。认真贯彻落实中央关于改进工作作风、密切联系群众的八项规定，大力开展以“为民、务实、清廉”为主要内容的群众路线教育实践活动。加快转变政府职能，深入推进政企、政资、政事、政社分开。继续深化行政审批制度改革，加快政府综合信息平台建设，打造全省一流的政务环境。深入开展“治庸问责”活动，切实做到“三短一简”，大兴政务清新之风。健全领导干部联系基层、联系企业、联系群众制度。对基层、企业、群众的合理诉求，要带着感情、带着责任，依法办、设法办、快速办。

坚持受人民监督。自觉接受市人大及其常委会的法律监督和工作监督，自觉接受市政协的民主监督，认真办理人大代表建议和政协委员提案，虚心听取和采纳民主党派、工商联、无党派人士的意见，自觉接受舆论监督和群众监督。深入推进政务公开，扩大公开范围，创新公开形式。坚持反腐倡廉与改革发展力度统一，严格执行党风廉政建设责任制，全面推进惩治和预防腐败体系建设，以反腐倡廉的实际成效取信于民。

时代赋予重任，人民寄予厚望。让我们紧密团结在以习近平同志为总书记的党中央周围，高举中国特色社会主义伟大旗帜，全面贯彻落实科学发展观，在省委、省政府和市委的坚强领导下，扎实工作，开拓创新，为实现绿色崛起、建设鄂南强市、打造“香城泉都”和“中三角”重要枢纽城市而努力奋斗！
